# Risk Factors for Revision After Early and Delayed Total Hip Arthroplasty Dislocation. An Analysis of Lithuanian Arthroplasty Register

**DOI:** 10.7759/cureus.14155

**Published:** 2021-03-28

**Authors:** Povilas Masionis, Tomas P Vileikis, Giedrius Kvederas, Valentinas Uvarovas, Igoris Šatkauskas, Tomas Sveikata, Jaunius Kurtinaitis

**Affiliations:** 1 Centre of Orthopedics and Traumatology, Vilnius University Hospital Santaros Clinics, Vilnius, LTU; 2 Centre of Orthopedics and Traumatology, Faculty of Medicine, Vilnius University, Vilnius, LTU; 3 Centre of Orthopedics and Traumatology, Republican Vilnius University Hospital, Vilnius, LTU

**Keywords:** tha, dislocation, revision, arthroplasty, register

## Abstract

Introduction: Despite relatively low incidence, dislocation remains one of the main reasons for total hip arthroplasty (THA) revision. It is a devastating complication for a patient and a surgeon, and has high burden on the healthcare system. The aim of the present study was to assess and compare the risk factors for revision after early and delayed THA dislocations.

Methods: Some 3403 THA through posterior approach for primary osteoarthritis were retrospectively studied in the Lithuanian Arthroplasty Register from 2011 to 2018. Three months after THA was the splitting time between the first event of early and delayed dislocations. Revision was set as outcome measure. Gender, affected side, number of dislocations, femoral head and neck size, and prosthesis fixation type were tested as risk factors for revision after early and delayed THA dislocations.

Results: Dislocation occurred in 108 patients (3.2%), and 26 cases (0.8%) required revision. Men had statistically significant higher risk for revision due to early dislocation [hazard ratio (HR) 4.7; 1.3-17.7 confidence interval (CI)] and considerably lower risk for revision due to delayed dislocation (HR 0.5; 0.1-1.7 CI). The left side THA had twice the risk as compared to the right in the early settings (HR 2.1; 0.6-6.9 CI) which equalized after three months (HR 1.1; 0.4-3.1 CI). Some 32 mm femoral head had significantly lower risk in the early group as compared to 28 mm head (HR 0.3; 0.1-0.5 CI). Short head was associated with increased risk for revision after early dislocation, although, not statistically significant. Prosthesis fixation type was not a risk factor for revision surgery neither after early nor after delayed dislocation.

Conclusion: The unique finding of gender separation was found -- men tend for revision after early dislocation and women after delayed dislocation. In early stage, additional precautions should be considered when 28 mm short metal heads are used.

## Introduction

With an aging population and growing demand for improved mobility and life quality in the increased cases of arthritis, joint replacement surgery is believed to become the most common elective surgical procedure in the following decades [1]. It is indicated in the United States that the number of patients with total joint replacement is similar to the number of patients with public’s attention catching chronic diseases, such as stroke or myocardial infarction and that the prevalence of total joint replacement is considerably higher than heart failure [1]. Dislocation in total hip arthroplasty (THA) is one of the most common reason for revision and has the incidence from 0.3% to 10% [2-3]. It is the most common cause of revision in the United states and the second after aseptic loosening in Swedish and France [2, 4]. A similar situation can be seen in Lithuania, where 66.2% of all revisions after THA are performed due to recurrent dislocations [5]. It is a devastating complication for a patient and a surgeon and has a high burden on the healthcare system [6]. Prevention of dislocation starts with thoughtful preoperative planning and assessment, surgical precision, and good postoperative care. However, about 60% of dislocated THA will relapse and 50% will require revision surgery [7]. If great trochanter is not significantly displaced, there is no visible component malposition or failed closed reduction, revision surgery is considered after two or even three dislocation episodes [8]. Risk factors for THA to dislocate are well known and classified to patient, surgeon and implant related, but risk factors for revision after dislocation remain unknown. Therefore, aim of the present study was to assess the risk factors for revision after early and delayed dislocations after THA.

## Materials and methods

**Figure 1:**
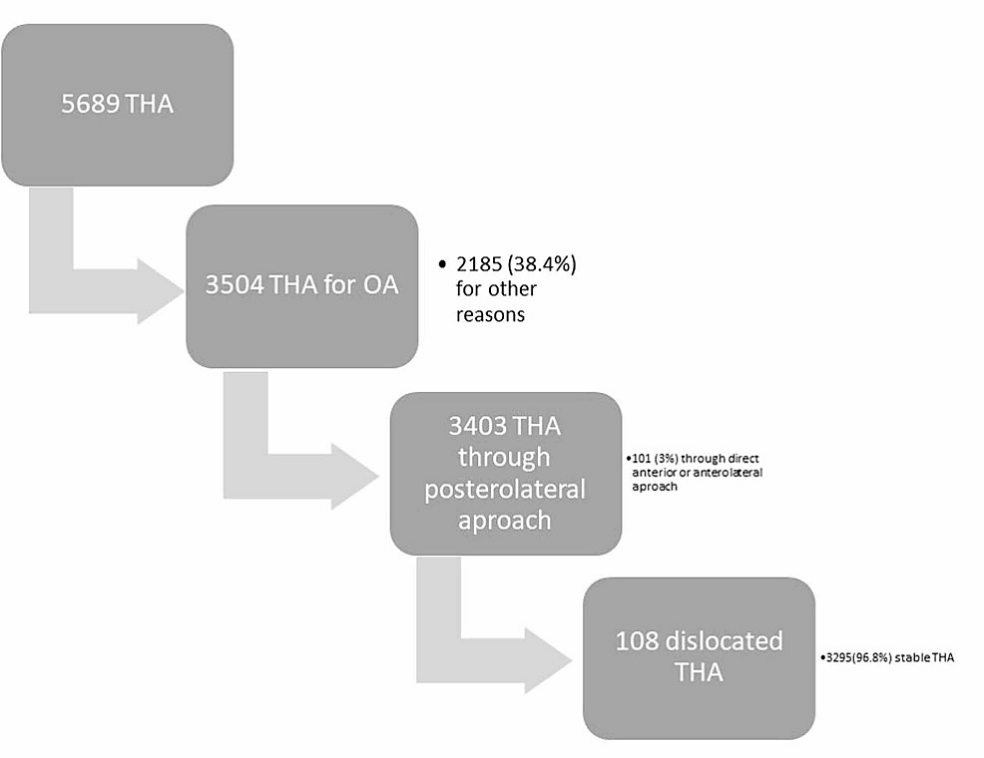
Flow diagram of patient exclusion and the final sample size.

Data were extracted from the Lithuanian Arthroplasty Register and included the period from January 1, 2011 to December 31, 2018. Some 5689 patients who went through THA were retrospectively studied. All patients, who were involved in a study, underwent primary THA through posterolateral approach (described by Moore) for primary arthrosis in the single institution [9]. Exclusion criteria were: revision THA, THA for femoral neck fracture, and stable THA. THA through direct anterior and direct lateral approaches were excluded because of the low sample size and the absence of dislocations. Patients who underwent surgery with the implant heads of a rarely used diameter (24, 26, 30, and 40 mm) were not included in the study. Cases of dual mobility or constrained cup were not included. Excluding patients according to above mentioned criteria, our final sample size was 108 patients (Figure 1).

Experienced radiologist evaluated THA X-rays (in cases of revision prerevision X-rays) for the following criteria: cup anteversion and inclination, femoral offset difference, and leg length discrepancy (LLD). The patient demographics and radiological parameters according to study groups are presented in Table 1. The patients were distributed according to the normal distribution by gender, age, side which was operated, and radiological parameters. In the study, the term ‘revision’ was defined as an open intervention where the whole prosthesis was eliminated, where an augmentation device was added, or where one or more parts of the implant were exchanged. Dislocations after THA (first occurrence) were divided into early and delayed. Some 90 days after THA was the splitting time between the incidence of early and delayed dislocations [10].

**Table 1:** Comparison of demographics and radiological parameters between early and delayed dislocation groups. LLD, leg length discrepancy

Variables	Early dislocation group (N = 43)		Delayed dislocation (N = 65)		p value
Gender (male/female)	17	26	23	41	0.66
Age (years)	68 ± 11		67 ± 10		0.63
Operated side (right/left)	25	18	44	21	0.31
Cup anteversion (degrees)	23.97 ± 8.37		21.37 ± 7.59		0.13
Cup inclination (degrees)	46.94 ± 9.30		46.63 ± 7.05		0.86
Femoral offset difference (mm)	0.28 ± 5.35		0.81 ± 7.98		0.73
LLD (mm)	3.21 ± 7.90		2.66 ± 8.73		0.77

Follow-up of patients began on the day when primary THA was performed and ended on the day of revision, emigration, death or December 31, 2018, whichever came first. Where appropriate median values, ranges, means, and 95% confidence interval (CI) were used by continuous descriptive statistics. Cox regression models were used to generate hazard ratio (HR) and its 95% CIs for various covariates, such as gender, head type, head size, head length, fixation method, and affected side. The level of signiﬁcance was set at p < 0.05 in all analyses and we used IBM SPSS 22.0 (IBM Corp, Armonk, NY, USA) statistical package for data analysis.

## Results

At follow-up out of 3403 primary THA dislocation occurred in 108 patients (3.2%), of which 26 cases (0.8%) had been revised. The first dislocation in 65 cases (60.2%) occurred later than three months after THA, while 43 (39.8%) dislocations occurred during the first three months. Some 14 (32.6%) early dislocations and 12 (18.5%) delayed dislocations required revision. The mean time from THA to revision surgery in the early dislocation group was 31.1 ± 21.2 days and 2.7 ± 2 years in delayed group. No statistically significant difference was observed in early and delayed dislocation groups in terms of degree of cup anteversion (23.97 ± 8.37 and 21.37 ± 7.60, p = 0.13 respectively), the degree of cup inclination (46,94 ± 9,30 and 46,63 ± 7,05, p = 0.86), the difference of femoral offset between both legs in millimeters (0.28 ± 5.35 and 0.81 ± 7.98, p = 0.73), and LLD (3.21 ± 7.90 and 2.66 ± 8.73, p = 0.77).

By entering all mentioned covariates into a multiple regression model, adjusted HRs were calculated (Table 2). In the Cox regression analysis, risk of revision due to early dislocation was statistically significantly higher in males than in females (HR 4.7; 1.3-17.7 CI, p = 0.02), while risk of revision due to delayed dislocation was considerably lower in men (HR 0.5; 0.1-1.7 CI, p = 0.26). The risk of revision after early dislocations of the left side THA was twice as high as the risk of the right side (HR 2.1; 0.6-6.9 CI p = 0.22), even though the risk equalized after three months (HR 1.1; 0.4-3.1 CI p = 0.84). We found a statistically significantly lower risk of revision due to early dislocation when using 32 mm head diameter than compared to 28 mm head [HR 0.3; 0.1-0.5 (CI p = 0.04)] and that ceramic heads in group of patients with early dislocation were associated with reduced risk of revision in comparison to metal heads [HR 0.6; (0.1-2.8 CI p = 0.51)]. An increasing head length of an implant also showed a reduction in risk of revision after early dislocation, with the lowest risk being observed when using long heads [0.3; (0.5-1.9 CI) p = 0.21], however, this observation was not statistically significant. Finally, we did not find any statistically significant evidence that the type of fixation (cemented or uncemented) affected the risk of revision after early [1.1; (0.2-4.9 CI) p = 0.95] or late [1.2; (0.3-4.4 CI) p = 0.76] dislocation. 

**Table 2:** HR, 95% CI, and p values of different factors for revision after early and delayed dislocation. HR, hazard ratio; CI, confidence interval

Group factor		Revision after early dislocation			Revision after delayed dislocation		
		HR	CI	p value	HR	CI	p value
Gender	Female	1			1		
	Male	4.7	1.3-17.7	0.02	0.5	0.1-1.7	0.26
Number of dislocations		1.2	0.8-1.8	0.49	1.3	1.0-1.7	0.06
Head type	Metal	1				1	
	Ceramic	0.6	0.1-2.8	0.51	1.0	0.3-3.2	0.99
	Head size	28 mm	1			1		
32 mm		0.29	0.1-0.5	0.04	0.9	0.3-2.8	0.83
36 mm		0			1.3	0.1-11.6	0.83
Fixation		Cemented	1			1		
	Uncemented	1.1	0.2-4.9	0.95	1.2	0.3-4.4	0.76
	Head length	Short	1			1		
Medium		0.8	0.2-3.0	0.69	2.1	0.5-9.9	0.34
Long		0.3	0.5-1.9	0.21	1.0	0.2-5.6	0.98
Affected side		Right	1			1		
	Left	2.1	0.6-6.9	0.22	1.1	0.4-3.1	0.84

## Discussion

The purpose of this paper was to estimate, whether revisions after early and delayed dislocations have the same risk factors, using data from the Lithuanian Arthroplasty Register -- gender and femoral head size were found as statistically significant factors separating revision risk after early and delayed dislocations.

The overall dislocation rate in our study was 3.2%, which is comparable to other reports [2-3]. Moreover, similar dislocation rate was found in the study by Woolson et al., in which 10,500 THAs were performed and the incidence in Italy is studied to be from 0.3% to 10% [11-12].

After adapting for THA and patient characteristics, our analysis shows that male gender is related to a significant higher risk of revision after early dislocation and considerably lower risk of revision due to delayed dislocation after THA. In literature we found little evidence about gender as a risk factor for revision after dislocation. An article by Hailer et al., stated that males have a higher risk for revision after dislocation after THA, but there was no distinction between early and delayed dislocations [4]. Recently, Rowan et al. wrote that neither of sex, simultaneous bilateral THA, or restrictive postoperative precautions have an impact on dislocation rates after THA [13]. We could not find any literature about the risk factors for revision after early and delayed dislocations, therefore, this finding is unique.

We did not find any literature about the influence of the operated side on the risk for revision. Even though we saw a tendency that the risk for revision after early dislocations of the left side was twice as high as the risk of the right side, our finding was not statistically significant. There is no literature on THA and dominant leg, but there are some reports on muscle strength difference and its clinical implication of dominant leg [14-15]. In our opinion, the impact of dominant leg on total joint arthroplasty outcomes is a hypothesis for further studies.

We chose a three-month period as the distinguishing point between early and delayed dislocations, because dislocations usually occur within a period of three months after THA. Up to 70% of dislocations occur during the first month after surgery or up to 66% occur during first five weeks [16-17]. Dislocations that happen within 0-3 months from surgery, usually occur due to patient factors, deficiency of mature scar tissue or tension in soft tissue, while delayed dislocations are most often caused by component malposition or polyethylene wear [10]. A study by Peters et al. shows that 93% of orthopedic departments in the Netherlands use patient restrictions following posterolateral approach THA [18]. In our clinic, the restriction period and rehabilitation process after posterolateral approach THA lasts for three months. Similar recommendations are described by Zahar et al., that for patients after THA rotation, flexion over 90° and adduction of the hip should be limited by the brace for six weeks, after that each motion modality should be gradually increased, while internal rotation and adduction should still be avoided for three months after operation. Therefore, dislocations that occurred after the end of rehabilitation period were considered delayed [19].

Our findings that head diameter of 32 mm is associated with lower risk of revision after early dislocation as compared to 28 mm head diameter are similar to what was stated by Conroy et al. and Girarg et al., that the increase of head size reduces the risk of revision [20-21]. Furthermore, we only analyzed THA done through posterolateral approach -- direct anterior and lateral approaches were excluded because of absence of dislocations and low sample number. Further, Pedneault et al. found that attention to surgical technique with posterior capsular closure outweighs the importance of femoral head size through posterolateral approach [22]. Although, the Lithuanian register of joint arthroplasty does not account the fact of whether the posterior capsule was reconstructed or not. Because of this reason, the question remains if the revision after early dislocation is associated with smaller femoral head or with unreconstructed posterior capsule.

In this study, there are some potential limitations that should be considered. First of all, it is a single institution experience extracted from the National Arthroplasty Register and it might be questionable if the results could be applied nationwide. Second, in our analysis we could not adjust for such variables, as patient BMI, activity levels, comorbidities, neurological disability, prosthetic malposition, implant impingement, hip anatomy restoration, alcohol abuse and mental status, and therefore could not assess patient demand on the implant. These variables were not available from the Lithuania Arthroplasty Register.

## Conclusions

The gender separation was found -- men tend for revision after early dislocation and women after delayed dislocation. The risk for the revision after early dislocation is twice higher when left hip was operated, although, clinical implication of this finding remains unclear. In early stage, additional precautions should be considered when 28 mm short metal heads are used.
